# The genetics of autism and steroid-related traits in prenatal and postnatal life

**DOI:** 10.3389/fendo.2023.1126036

**Published:** 2023-05-08

**Authors:** Alex Tsompanidis, Varun Warrier, Simon Baron-Cohen

**Affiliations:** Autism Research Centre, Department of Psychiatry, University of Cambridge, Cambridge, United Kingdom

**Keywords:** autism, placenta, neuroplacentology, genetic correlation, menarche age, sex difference

## Abstract

**Background:**

Autism likelihood is a largely heritable trait. Autism prevalence has a skewed sex ratio, with males being diagnosed more often than females. Steroid hormones play a mediating role in this, as indicated by studies of both prenatal biology and postnatal medical conditions in autistic men and women. It is currently unclear if the genetics of steroid regulation or production interact with the genetic liability for autism.

**Methods:**

To address this, two studies were conducted using publicly available datasets, which focused respectively on rare genetic variants linked to autism and neurodevelopmental conditions (study 1) and common genetic variants (study 2) for autism. In Study 1 an enrichment analysis was conducted, between autism-related genes (SFARI database) and genes that are differentially expressed (FDR<0.1) between male and female placentas, in 1^st^ trimester chorionic villi samples of viable pregnancies (n=39). In Study 2 summary statistics of genome wide association studies (GWAS) were used to investigate the genetic correlation between autism and bioactive testosterone, estradiol and postnatal PlGF levels, as well as steroid-related conditions such as polycystic ovaries syndrome (PCOS), age of menarche, and androgenic alopecia. Genetic correlation was calculated based on LD Score regression and results were corrected for multiple testing with FDR.

**Results:**

In Study 1, there was significant enrichment of X-linked autism genes in male-biased placental genes, independently of gene length (n=5 genes, p<0.001). In Study 2, common genetic variance associated with autism did not correlate to the genetics for the postnatal levels of testosterone, estradiol or PlGF, but was associated with the genotypes associated with early age of menarche in females (b=-0.109, FDR-q=0.004) and protection from androgenic alopecia for males (b=-0.135, FDR-q=0.007).

**Conclusion:**

The rare genetic variants associated with autism appear to interact with placental sex differences, while the common genetic variants associated with autism appear to be involved in the regulation of steroid-related traits. These lines of evidence indicate that the likelihood for autism is partly linked to factors mediating physiological sex differences throughout development.

## Introduction

Autism is substantially heritable, with twin based heritability estimates ranging from 64 – 93% (1–3). Exome sequencing studies have been successful in uncovering many syndromic forms of autism that are attributed to protein truncating or missense variants in constrained genes, most of them *de novo*, including both copy number variants and single-gene mutations (4). The Simons Foundation for Autism Research (SFARI) has collected information on all of these genes, compiling curated lists, based on available evidence (5). These are scored according to the number of “hits” across all studies, the strength of the evidence for each and whether these are involved in syndromic or non-syndromic forms of autism. Taken together these identified genes account for less than 10% of the variance in autism, with the majority of the variance in autism being due to common genetic variance (6, 7). In order to discover specific genes, two related case-control genome-wide association studies (GWAS) have been conducted (8),(9). The largest included n=18,381 autistic people diagnosed in Denmark who were linked to a validated diagnosis via the National Psychiatric Register and were genotyped as part of iPSYCH (9). This study led to the discovery of five genome-wide statistically significant loci or single nucleotide polymorphisms (SNPs). Grove et al. (9) estimate that a considerably larger sample would be needed to uncover more SNPs and their related molecular pathways. As with all complex heritable traits, genetic variants of intermediate frequency will also need to be discovered via sequencing, in order to bridge the gap between common polygenic and rare monogenic causes of autism (10).

However, it remains unclear how this largely genetic condition may affect males and females differently and lead to different prevalence rates for each sex. Two hypotheses have been put forward to resolve this (11). First, that autism in males and females may be caused by different genetic factors, which functionally converge at the neuronal or only at the behavioral level, leading to different likelihood distributions for each sex. Second, that autism in both males and females is caused by the same genetic variants but underlying physiological sex differences interact with genetic likelihood and result in a sex-specific threshold for diagnosis, within the same likelihood-threshold model. In accordance with this, exome sequencing studies have consistently found that autistic females have a larger number of loss-of-function mutations, compared to males (4, 12, 13), although, recent work has suggested that this is primarily limited to a few genes associated with severe developmental disorders (14). Hormonal factors (such as prenatal steroids) or differences in physiology (such as differing growth rates) may be mediating this excess of PTV mutations in autistic females compared to males (15, 16).

At the molecular level, the interaction between autism genes and sex may be bidirectional. Autism-related genetic variants may be directly affecting sex differentiation mechanisms, including steroid hormone levels, leading to different effects for each genetic variant in males and females. Alternatively, steroid hormones may be involved in the regulation of the same autism-related genes, resulting in relative differences in how their gene products are expressed and translated in males and females.

With regards to the first of these hypotheses, candidate gene studies have reported associations with autism and autistic traits in polymorphisms in hormone receptors (e.g. the estrogen receptor ESR2), as well steroid-regulating enzymes (e.g. aromatase) (17, 18). But these findings have not been confirmed in GWAS (9). With regards to the second hypothesis of a mediatory role of sex steroids, a study of testosterone administration in developing neurons found that the genes affected by the hormone (identified via ChIp-Seq) significantly overlap with autism-related genes, including SFARI genes and genes that are differentially expressed in the autistic brain (identified by RNA-Seq post-mortem) (19). However, a wider comparison of all the genes that have steroid-responsive promoters failed to find any enrichment. Instead, a moderate correlation of autism-related genetic variants was reported with genes relating to sex differences in anthropometry (e.g. height) (20). The same study reported sex heterogeneity with regard to X-chromosome-linked genetic variants associated with autism. Genetic variants on the X-chromosome could also be mediating the reduced prevalence of autism in females, particularly in genes that escape inactivation or are characterized by haploinsufficiency when mutated. However in autism, studies have again produced mixed results regarding a significant enrichment for X-linked genes, or more specifically in regions that escape X-chromosome inactivation (21–24).

Thus, there is little evidence that autism genes directly regulate steroids or lie directly downstream of steroid-responsive elements. Nevertheless, the functional interaction between steroid hormones and autism genetics may be indirect and further downstream, at the level of molecular pathways and networks. In addition, increased male likelihood for autism may be mediated by factors that are not specific to autism and that also extend to other neurodevelopmental conditions that show sex differences in prevalence. Consistently, a study that analyzed genetic variants associated with autism, together with ADHD(attention deficit hyperactivity disorder), anxiety and other co-occurring traits, reported that their combined downstream targets and associated molecular pathways converged on networks that feature prominent roles for the estrogen receptor (ER-2), as well as aromatase (25). Moreover, the interaction between autism genes and sex differentiation processes may be specific to early development, embryonic pathways, and particularly prenatal physiology. As previously mentioned, one of the few studies to report an overlap between testosterone targets and autism-related genes, focused their enrichment analysis on developing neurons (19). However, steroid-synthesizing tissues, such as the placenta, have not yet been analyzed in a similar way.

There are several lines of evidence that support a role for the placenta in neurodevelopment and specifically in autism. Independent studies have shown that sex steroids are elevated prenatally in autistic males compared to non-autistic males and that this effect is more apparent for estrogens; the majority of which are produced prenatally by the placenta (16, 26, 27). A similar observation has been reported with regard to autistic traits in infancy (28). In the last five years, it has also become clear that the likelihood of autism in the children is increased if the mother has been diagnosed with polycystic ovaries syndrome (PCOS) (29–31) and this is more specific to the hyperandrogenic form of the condition (32). Interestingly, PCOS is also associated with changes in placental steroid synthesis, such as decreased aromatization (33, 34). Furthermore, placental complications have also been associated with autism in several independent epidemiological studies (35–37), but the molecular mechanisms that may be driving this are unclear. Atypical placental morphology and vasculature in autism have been reported in smaller clinical studies (38), together with increased placental inflammation and size (39, 40). On the genomic level, different patterns of placental DNA methylation have been observed in fetuses later diagnosed as autistic (41), with differential methylation enriched for promoters of genes associated with autism (4) and genes that are dysregulated in autistic brains post-mortem (42).

More importantly, placental function is sexually dimorphic, with placentas of male fetuses being more vulnerable to early complications (43), producing more steroids (44) and more factors associated with placental hypertension, such as PLGF (placenta growth factor), which activates angiogenic pathways in response to complications (45). Interestingly, PLGF levels in maternal serum can also predict autistic traits and mediate higher autistic traits in malesof the same cohort compared to females (46). Taken together, these findings support the hypothesis that sex differences in the placenta may partly be mediating sex differences on autism likelihood.

In addition, if autism genes and steroid-related molecular pathways interact beyond prenatal development, then this may also affect health outcomes throughout life and lead to higher prevalence of steroid-related symptoms and conditions in autistic people. Although, epidemiological evidence is consistent with this, particularly for conditions such as PCOS (30, 47, 48), the association between autism and steroid-related conditions has not been specifically shown at the genetic level.

In order to better understand the interaction between sex and genetics in autism, two exploratory analyses are presented in Studies 1 and 2. These respectively pertain to rare and common variants as well as prenatal and lifelong outcomes. In Study 1, genes that show sex differences in placental expression are tested for enrichment for ‘high-confidence’ autism genes that have been consistently implicated in autism by exome sequencing or in syndromic forms of the condition (SFARI categories 1 and S). In Study 2, autism-related common genetic variance (captured via GWAS) is studied in association with the genetic variants relating to steroid hormone levels in middle-aged or older adults (UK Biobank) and of the placental growth factor (PlGF), as well as to the genetics of complex steroid-related traits (publicly available data on age of menarche, PCOS and androgenic alopecia).

## Methods

### Study 1 - Rare variance: enrichment of autism genes in male placentas

Prenatal gene expression differences between the placentas of males and females were reported in a study by Gonzalez et al. (n=17 females, n=22 males)(49). To our knowledge, this is the only study that has investigated these differences in human pregnancies that were carried to term and at a time-point corresponding to 10.5 - 13.5 weeks gestation. This was achieved with RNA sequencing of discarded chorionic villi samples following prenatal genetic screening of the pregnancies, which were all carried to term. No additional information was reported for the clinical reasons that led to CVS(chorionic villi sampling). The same placentas were then collected after birth and their gene expression was compared to measurements during gestation. Other than birth weight, cohort covariates such as maternal age or crown rump length did not significantly differ between the sexes. Sex chromosomes were analyzed separately to autosomes and were found to contribute significantly to gene expression differences between the sexes. This was mainly driven by genes on the X chromosome and remained consistent throughout gestation and at birth. In contrast, most of the autosomal genes with sex differences in the 1^st^ trimester samples did not show the same differences when placentas were assessed at birth (49). This study was chosen over other reports of placental gene expression at birth or following elective termination, to minimize confounding due to labor onset and to better capture placental transcriptome following the prenatal masculinization window in males (50).

For the purposes of the current enrichment analysis, a list of genes was created that correspond to the transcripts identified by Gonzalez et al, as being significantly different between the sexes (both up- and down-regulated), at an FDR-corrected level of significance of q<0.1. This list comprised of n=135 genes, n=31 of which are on the X chromosome. Regarding autism, a list of high-confidence genes was assembled, based on the curated database of SFARI. Only genes with the top confidence score of “1” (n=206), as well as “syndromic” genes were included (n=143). Based on SFARI policy, the former score is only awarded to genes with a minimum of three independent discoveries in sequencing studies and a likely loss-of-function effect (4). The latter characterization (‘syndromic’) refers to genes of identified syndromes that also substantially increase the likelihood for autism. The two categories are not mutually exclusive, with the final list that was checked for enrichment numbering n=286 different autism-related genes, of which n=34 are on the X chromosome.

Enrichment analysis was conducted separately for autosomes and the X chromosome, by comparing the number of genes overlapping between the two lists (autism and placenta), to the total number of coding genes in the genome. Logistic regression models were used, in order to control for gene length as a covariate in these comparisons. Information on identified coding genes, gene locations and lengths were obtained from Gene Ensembl.

### Study 2 - Common genetic variants: genetic correlations between autism and steroid-related traits

For the purposes of genetic correlation analyses, publicly available summary statistics of GWAS were collected, regarding postnatal hormone levels and hormone-related traits. Datasets for these were obtained via the NHGRI-EBI Catalogue (**Table 1**).

**Table 1:** GWAS of hormone levels and steroid-related traits that were investigated in association with autism.

	Bio T	Bio T	Estradiol	PlGF	PCOS	Age of menarche	Alopecia
***Study***	51			52	53	54	55
***Cohorts***	UK-Biobank			various, SCALLOP	various in UK, NL, USA	ReproGen, 23andMe, UKB	UK Biobank
***N***	178,782	188,507	206,927	21,758	24,267	329,345	52,874
***Sex***	males	females	males	both	Female cases, mixed controls	females	males
***Age***	40 - 69	40 - 69	40 - 69	>40	27 - 60	NA	40 - 69
***Loci***	125	147	22	2	14	389	250

Bio T: free fraction of circulating testosterone, based on testosterone and SHBG.

In terms of neurodevelopment, two outcomes were selected. First, clinically diagnosed autism, based on the latest GWAS (n=46,350 (18,381 cases))(9), that identified five loci at genome-wide level of significance and corresponded to both males and females with the condition, as identified and confirmed by the iPSYCH consortium, as well as the Psychiatric Genomics Consortium that had previously published the first autism GWAS (Psychiatric Genomics Consortium 2017). The second outcome that was investigated corresponded to the entirety of the iPSYCH cohort and included some of the same autistic individuals (n=6,939 overlap in cases), as well as anyone with ADHD, affective disorder, bipolar disorder, anorexia, or schizophrenia, who were diagnosed and genotyped in Denmark (total n=65,534 including controls) (56). Therefore, the second trait outcome included autism (26.2% of iPSYCH are autistic) but also individuals with conditions that often co-occur with autism. This second aggregate outcome was included in this study in order to increase power in correlation analyses and investigate the specificity of potential findings.

Regarding hormone levels and steroid-related conditions, summary statistics of several GWAS were obtained online via publicly available resources (**Table 1**).

Bioavailable testosterone was calculated in the UK Biobank, by dividing total testosterone levels to the concentrations of SHBG for each individual (51). This was calculated for both males and females and then linked to genotype data. In the same cohort, estradiol level measurements were available in both genders, but the GWAS for estradiol levels was restricted to males, as no heritable component corresponded to this trait was found in females. This was potentially due to low trait variance, as the cohort consisted of mainly post-menopausal women whose estradiol levels were below the level of detection (78% of the female cohort).

Placental growth factor (PlGF) levels were measured during adulthood, in circulating serum, as part of proteomic analyses that were aggregated into a meta-analysis of different studies (the SCALLOP consortium) that investigated associations of serum biomarkers with health outcomes. In this study, circulating PlGF levels were significantly associated with the genetics of cardiovascular disease, an effect that the authors attributed to the angiogenesis role of PIGF, which extends beyond pregnancy (52).

The GWAS for PCOS included data from clinically diagnosed cases, as well as self-report cases from the company 23andMe, resulting in many genome-wide significant loci that were linked to steroid hormone regulation and gonadotropin regulation, independently of the method of diagnostic ascertainment (53). For the purposes of this study, self-report data on PCOS were excluded and genetic correlation was restricted to clinically ascertained cases of the condition. The GWAS on age of menarche, on the other hand, included self-report genotype data from 23andMe, meta-analyzed together with data from the UK Biobank and the Reproductive Genetics Consortium (54).

Finally, androgenic alopecia was ascertained in adult males in the UK Biobank, who are older than 40 years old. The trait was analyzed categorically, based on the degrees of hair loss and age of onset. The X chromosome was analyzed separately to the autosomes, given the established and disproportionate role of the X-linked androgen receptor gene, and was therefore not included in the summary statistics for this trait (55).

Genetic correlation of common genetic variants was based on LD score based regression using LD scores in the north-west European populations (57). A breakdown of sample size for each SNP was included, where available. If SNP identifiers were missing, these were derived based on SNP location and the NCBI-SNP database. Multiple testing correction was conducted with application of a false discovery rate (FDR), based on the number of tests (n=7) for each neuropsychiatric outcome separately.

## Results

There was no overlap between autosomal genes that were differentially expressed in male and female placentas, and high-confidence autism genes. The comparison that was specific on the X-chromosome identified a total overlap of n=5 genes (**Table 2**). This was significant in a logistic regression model that controlled for gene length and compared this to the total number of genes on each list (n=34 for autism, n=31 for the placenta) and on the X-chromosome. The model showed that this enrichment was significant at p<0.0001, for an OR=11 (3.6 - 33.7). All five of the genes were differentially expressed in placentas at FDR-corrected p<0.05, with four being upregulated in females, compared to males (**Table 2**).

**Table 2:** SFARI Genes that show significant sex differences in placental expression.

Gene ID	Gene Name	M/F	Autism Total	Gene Function	SFARIScore	Conditions
***SMC1A***	structural maintenance of chromosomes 1A	Female upregulated	2/8	Segregation of sister chromatids during division	S	Cornelia de Lange - 2, DD/NDD, EPS Rett-like phenotypes
***KDM5C***	lysine demethylase 5C	Female upregulated	6/27	transcription and chromatin remodeling	1	EPS, Autism, EP, DD/NDD
***ARHGEF9***	Cdc42-guanine nucleotide exchange factor (GEF) 9	Male upregulated	4/11	Rho-like GTPase, mTOR regulator	1S	EPS, ID, Autism
***HDAC8***	Histone deacetylase 8	Female upregulated	1/8	suppresses transcription in large multiprotein complexes	S	Cornelia de Lange - 5
***DDX3x***	DEAD box helicase 3	Female upregulated	7/27	transcription regulation, mRNA assembly, splicing	1S	DD/NDD, ADHD, EPS, ID, Autism, EP

**Figure 1:**
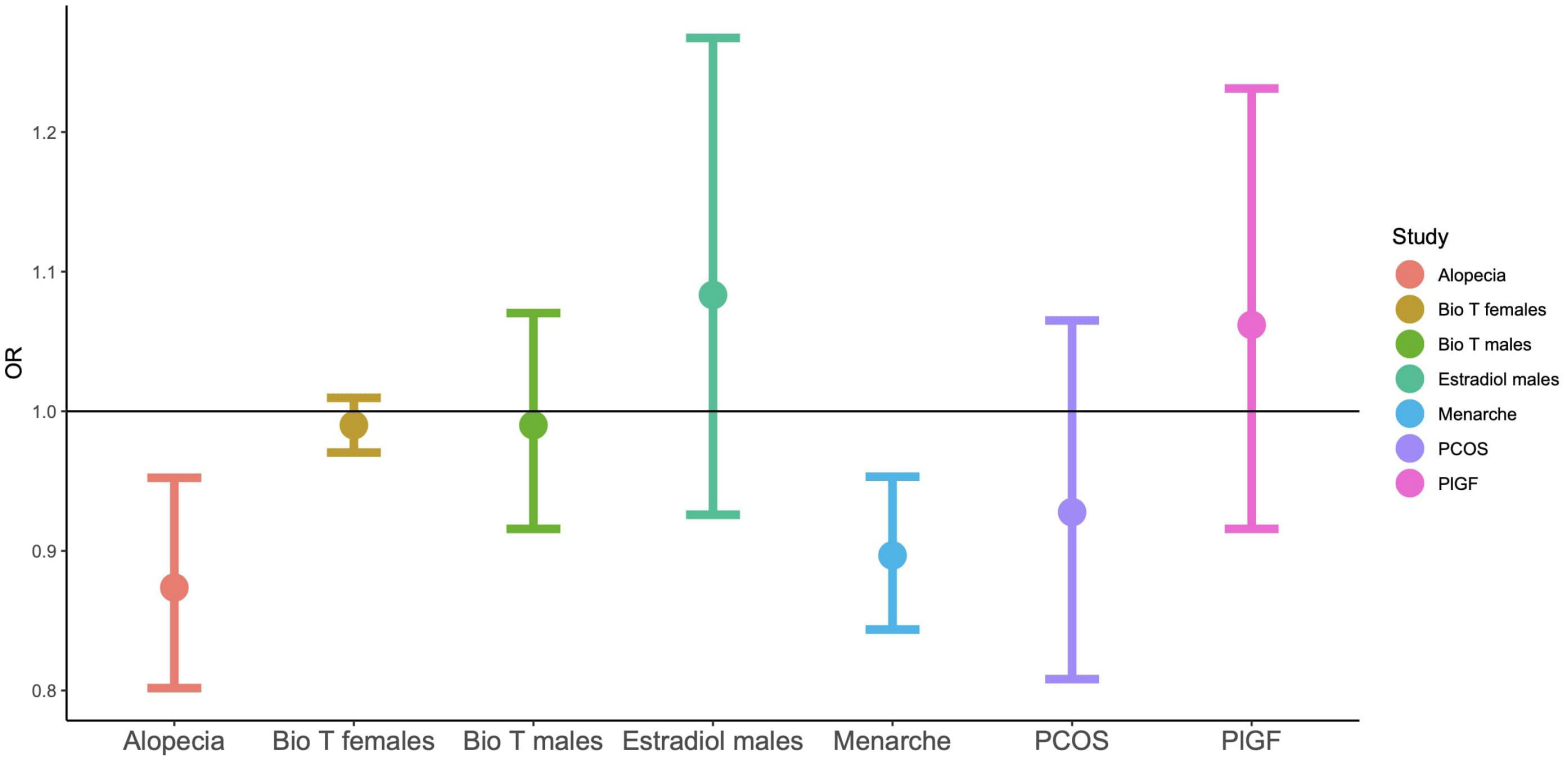
Odds ratios for LD-score regressions for autism based on GWAS summary statistics. ‘Bio T’: the levels of free testosterone, based on circulating total testosterone and SHBG.

In Study 2, of all the examined hormones and traits, significant genetic correlations were identified between autism and age of menarche and alopecia (**Table 3**, in bold). The former correlation extended to the wider iPSYCH dataset that included additional psychiatric conditions, while the latter failed to reach statistical significance in this wider category, after controlling for multiple testing. Both of the genetic correlations were negative in direction, with higher genetic likelihood for autism being associated with an earlier age of menarche and reduced likelihood for hair loss (**Figure 1**).

**Table 3:** Pairwise genetic correlations based on LD scores of GWAS summary statistics. q is FDR-corrected value of statistical significance.

	Bio T males	Bio T females	Estradiol males	PLGF	PCOS	Age of menarche	Alopecia
	h2: 0.13 (0.01)	h2: 0.16 (0.01)	h2: 0.02 (0.01)	h2: 0.12 (0.05)	h2: 0.13 (0.02)	h2:0.21 (0.01)	h2: 0.30 (0.05)
***Autism*** *h2: 0.19 (0.02)*	b=-0.01	b=-0.01	b=-0.06	b=0.06	b=-0.075	**b=-0.109**	**b=-0.135**
	SD=0.04	SD=0.01	SD=0.08	SD=0.09	SD=0.084	**SD=0.031**	**SD=0.044**
	z=-0.25	z=-0.20	z=-0.78	z=0.61	z=-0.893	**z=-3.482**	**z=-3.077**
	p=0.80	p=0.84	p=0.438	p=0.55	p=0.372	**p=0.0005**	**p=0.0021**
	q=0.84	q=0.84	q=0.767	q=0.77	q=0.767	**q=0.0035**	**q=0.0074**
***iPSYCH*** ***combined*** *h2: 0.13 (0.01)*	b=-0.07	b=0.08	b=-0.01	b=-0.08	b=-0.021	**b=-0.124**	b=-0.106
	SD=0.04	SD=0.04	SD=0.07	SD=0.09	SD=0.09	**SD=0.036**	SD=0.044
	z=-1.61	z=2.01	z=-0.16	z=-0.85	z=-0.23	**z=-3.452**	z=-2.402
	p=0.11	p=0.045	p=0.09	p=0.39	p=0.818	**p=0.0006**	p=0.0163
	q=0.154	q=0.105	q=0.154	q=0.455	q=0.818	**q=0.0042**	q=0.057

‘Bio T’: the levels of free testosterone, based on circulating total testosterone and SHBG.

## Discussion

### Study 1 - Genetic overlap on the X chromosome

This analysis compared the gene expression differences in the placenta, during mid-pregnancy, to a list of genes that have been associated with autism. While there was no genetic overlap when autosomes were considered, there was a significant enrichment when the analysis was restricted to the X-chromosome. This was due to an overlap of five genes on the X, namely *SMC1A, KDM5C, ARHGEF9, HDAC8* and *DDX3x*. Four showed significant sex differences in placental expression at a level of FDR<0.01 (with the exception of *ARHGEF9* at FDR<0.05) indicating that enrichment would have been significant at more conservative thresholds than the one chosen for this study (FDR<0.1).

Sex differences in placental function and vulnerability have long been known to clinicians. However, the molecular background to this has only been studied relatively recently, following the discovery of high-throughput genomic methods, such as microarrays and next-generation-sequencing. Consistently, across all human studies, placental gene expression differences between the sexes can largely be attributed to differences in the dosage of genes, located on the X and Y chromosomes (44, 58). In the study by Gonzalez et al, prenatal differences in the expression of genes on the X chromosome were consistent with measurements conducted at birth. In contrast, differences in autosomes fluctuated according to gestational age (49). Further analysis showed that the placental X-linked genes that differed by sex were not restricted to regions that are known to escape X-chromosome inactivation, but rather extended across the length of the chromosome.

In a more recent, large-scale study of placental genomics (at birth), researchers were able to compare gene expression differences not only between the sexes, but also within the placentas of the same individuals. They also reported significant sex differences, stemming from the X chromosome. However, intra-individual comparisons, paired with genotyping, also revealed that there was pronounced genetic mosaicism within the same placentas, which extended to transcriptional differences (59). This complex genetic landscape can potentially be attributed to placental tissue’s clonal expansion and may be driven by rapid cycles of DNA replication and cell division. This could in turn lead to inefficiencies in chromosome methylation, X-chromosome inactivation and genetic instability, leading to accumulation of mutations confined within clonal cell populations, similarly to neoplastic growth. Thus, sex differences in placental genomics and ultimately function may be attributed to the tissue’s complex genetic landscape, gene dosage effects on the X-chromosome and this controlled state of genetic instability.

This phenomenon may also be affected by the effects of hormones and the person’s own genetic background. Male placentas express higher levels of DHEAS, the main steroid precursor for both androgens and estrogens (22). In the study by Gonzalez et al, unsupervised analysis of the identified variants via the Ingenuity software, identified the estrogen receptor-1 and progesterone as potential upstream regulators of the identified sex differences (49). It is also important to note that sex steroid hormones have pronounced effects on methylation, by directly acting on histone modification enzymes, as well as the genome itself (via their receptors)(60). Therefore, sex differences in placental gene expression are probably not independent to the effects of sex steroid hormones.

Regarding autism, prenatal steroidogenesis, methylation differences in the placenta and genetic instability have all been associated with increased likelihood for the condition (61). In this study, five genes were identified that may be contributing to the interactions between these factors.

All five of the genes take part in the regulation of genomic processes, such as transcription, gene replication and mitotic segregation. However, some have also been linked to steroid-related phenotypes. For example, *KDM5C* encodes a DNA-binding transcription regulator that is involved in chromatin remodeling. But a common genetic variant directly upstream of this gene (rs140498714) was identified in the largest GWAS of testosterone levels in older adults of the UK Biobank (51). Syndromes linked to the same locus have also been associated with abnormalities in the formation of the male external genitalia. The main example is the syndrome attributed to MRXSCJ (X-Linked, Syndromic, Claes-Jensen Type), which, in addition to neurodevelopmental symptoms, also features short stature, cryptorchidism, micropenis and endocrine symptoms in later life, like amenorrhea (in females), alopecia (in males) and excessive body hair growth in neonates and children (62). In addition, in a mouse knockout model for *Kdm5c*, there was a significant increase in the animals’ aggressive behavior. These changes were attributed to changes in the brain, where dendrite differences, an increase in spines and upregulation of androgen targets, were noted in the amygdala and frontal cortex (63).

Similarly, *DD3X* encodes a helicase involved in transcription and splicing, which has been linked to the main symptoms of Klinefelter syndrome. Individuals with this syndrome have increased prevalence for autism, but also frequently have defects in primary and secondary sex differentiation, with frequent eunochoid body proportions and primary hypogonadism (small gonads and decreased testosterone) (64, 65). In a mouse model of the condition, the homologous gene was seen to escape X-chromosome inactivation, leading to a significant up-regulation of the transcript in the brain of the Klinefelter-type, compared to the brains of both male and female mice (66). It is interesting to speculate that loss-of-function mutations in autism may have the opposite effect and lead to sex steroid excess. DD3X is also one of the genes that ‘escapes’ X chromosome inactivation in somatic cells of females as well as the placenta, potentially leading to gene dosage differences between the sexes (67).

Contrary to this and the other genes, which were downregulated in male placentas, the transcript for *ARHGEF9* was found to be upregulated in males. This gene encodes a guanine nucleotide exchange factor, that takes part in molecular cascades involving CDC42 and the mTOR pathway. In a clinical setting, *ARHGEF9*-related syndromes have been associated with autism, developmental delays and speech impairment, with epilepsy being a common symptom in most cases (68, 69). At the molecular level, the gene product is reported to contribute to the formation of GABAnergic and glycinergic synapses in the brain. Interestingly, common variance around the gene has also been linked to total testosterone levels (in men) (51), as well as male-pattern alopecia (within an intron of the gene) (55).

Finally, mutations in two of the genes that were identified in this analysis (*DDX3X, SMC1A*), can lead to Cornelia-de-Lange syndrome (CdLS). This rare genetic condition is characterized by mental and physical delays, microcephaly and a characteristic facial phenotype with short brows and synophrys (70). The children often exhibit autistic behaviors in response to overstimulation. This symptomatology is often worse in males, than females. Interestingly, children with the condition are often born with excess body hair, but this may be the result of dysfunctions in epithelial differentiation of the skin, rather than an example of hirsutism due to excess testosterone. Research into the endocrine correlates of the condition is limited, with an unpublished conference report describing significant differences in testosterone (71). At the molecular level, clinical sequencing in *CdLS* has revealed widespread defects in transcription and genetic instability (72).

All of these examples indicate a molecular link between genetic pathways involved in neurodevelopment, genetic instability and in some cases, sex differences and the endocrine system. However, caution should be employed in the interpretation of the observed enrichment, as the result may be confounded by the ‘X-factor’. Genes on the X chromosome have been found to be highly expressed in the placenta, as well as in the brain, relative to other chromosomes (73, 74). In line with the latter, there is also a relative overabundance of X-linked genes that are associated with mental disabilities (75), and X-linked common variance in the UK Biobank has recently been shown to be associated with neuroanatomical differences in many brain regions (76). Therefore, in the context of Study 1, the SFARI-curated list of genes may not be specific to autism, but rather reflect a link between the human brain and the X-chromosome. In addition, there is potential for clinical ascertainment bias in the study by Gonzalez et al, as the CVS samples pertained to pregnancies that were recommended for genetic screening. No clinical information is reported by the authors, other than relatively advanced maternal age (mean=39.4[SD=2.4]years old), so their genomic results may not be generalizable to prenatal placentas in the wider population.

In conclusion, this enrichment analysis offers a first indication that placental sex differences at the genetic level may be overlapping with the genetic likelihood for autism. More research is needed to study placental gene expression profiles that are specific to autism, as well the role of the autosomes.

### Study 2 - Genetic correlation analysis

The majority of the genetic likelihood for autism can be attributed to common variants (6). In this exploratory analysis, the common-variant genetics of autism was examined in association with the genetics of several steroid-related traits and conditions. It is important to note that these genetic correlations only capture the polygenic component of each trait, relating to common genetic variance. They are not tests of a link at the clinical level. In addition, each GWAS is affected by varying degrees of statistical power, as well as the specific demographic characteristics of each cohort. Particularly regarding steroid hormone levels and PlGF levels, their assessments in the corresponding GWAS were all postnatal and in late adulthood, setting them apart from the other studies that included prenatal measurements of the same hormones (16, 28, 77). This may be the reason that no genetic correlation was found between the genetic variance associated with testosterone, estradiol or PlGF levels with autism, despite previous clinical and epidemiological evidence. Alternatively, elevated steroidogenesis in autism may be due to genetic factors that are not captured well by the latest GWAS, but rather are related to rare variants, the X-chromosome or to interactions between genes and the wider endocrine system.

For example, polycystic ovaries syndrome (PCOS) is a complex and heterogeneous condition that includes multiple hormonal systems and has not yet been attributed to a single cause. In the context of autism, elevated rates of PCOS have been reported in autistic women (30). However, the evidence is certainly more conclusive for the effects of maternal PCOS on autism likelihood in their children, following replications in independent cohorts (78). In the present study, no genetic correlation was found between the genotypes associated with PCOS and the ones for autism. This could be an indication that the link between the two conditions may be attributed to ‘environmental’ factors, such as increased androgen exposure *in utero*. The placentas of women with PCOS have lower levels of aromatase, potentially exposing the fetus to elevated levels of maternal androgens (33). Consistent with this, new evidence in humans indicates that PCOS may be transgenerational, whereby high levels of prenatal androgens in mothers with the condition induce PCOS in their daughters via conditioning of the HPG(hypothalamus-pituitary-gonadal) axis, rather than via direct genetic effects (79). This could partly explain why the strong epidemiological link of PCOS with autism likelihood does not appear to be mediated by genetic factors in this analysis or in epidemiological studies (31).

In contrast, the present analysis showed that the genotypes associated with hair loss in men genetically correlate negatively with autism. The effect size is considerable (b=-0.14 [0.04], p=0.007) and higher than the genetic correlation of the same trait (androgenic alopecia) and with the genetics of bioavailable testosterone in men (b=0.10 [0.03], p=0.003). The direction of effect is surprising, given previous findings of elevated circulating androgens in autism postnatally (80). On the other hand, several studies from our lab (16, 28) and by other research groups (27) have reported that autism is associated with higher levels of estrogens, which could, in turn, have an effect on reducing androgenic alopecia likelihood postnatally. No previous clinical or epidemiological study has investigated whether androgenic alopecia is less frequent in autism, and most reports of symptoms relating to increased steroids have focused on women and their reproductive health (47, 48). If this genetic correlation extends to the clinical level, then this could be seen as additional evidence that a potential ‘steroidopathy’ in autism affects males and females in different ways. It is also important to note that androgenic alopecia is a complex, polygenic trait that likely involves several factors other than androgen levels/sensitivity, such as hair thickness, follicle vascularization and epithelial aging. Several studies have reported a genetic link between the androgenic alopecia and neurodegenerative conditions, such as Parkinson’s (81, 82). In the genetic correlation presented here, genotype data was restricted to the autosomes and did not include variance relating to the androgen receptor on the X-chromosome. Nevertheless, the result, albeit surprising, remains significant and likely captures a common latent factor in the maintenance of follicle health in men and the likelihood for autism.

Finally, the analysis showed that autism correlated negatively with age of menarche in women. This finding is consistent with previous epidemiological reports of a negative association between puberty onset and autistic traits (83), as well as higher likelihood for early onset of puberty in autistic people (84, 85). However, delayed puberty, in the context of hypogonadotrophic hypogonadism (e.g. in Klinefelter’s), has also been associated with increased likelihood for concurrent autism (OR=5.7 [2.6-12.6]), ADHD and other neurodevelopmental conditions (86).

Puberty onset, as well as age of menarche are both complex traits that feature an interplay between gonadal steroids, hypothalamic signaling, and pituitary rhythmicity. It is still unclear how autism affects the maturation of these components in childhood and puberty. Atypical rates of cortical thinning during adolescence have been reported for several brain regions in autistic individuals. In one study, the observed patterns of cortical thinning interacted with sex and were proposed to be mediated by an initial resistance to steroid-induced neuronal pruning, followed by an accelerated rate in later adolescence (87). Faster pubertal development has also been linked with increasing symptom severity for other aspects of mental health. Males and females who mature earlier than their peers report more symptoms of depression (88, 89) and are at increased risk of attempting suicide (90), with the rate of pubertal development being a particularly strong predictor of depression in males (89). A survey of over 35,000 Finnish teenagers showed that early onset of puberty was associated with internalizing (e.g. depression, anxiety, psychosomatic problems, bulimia) and externalizing behavior (substance-use, bullying, truancy) in females, but only the latter in males (91). These reports are also consistent with the genetic correlation that was observed in this study, between early puberty onset and the genetics of several psychiatric conditions (**Table 3**). Therefore, this link may not be specific to autism and likely relate to both genetic and environmental factors that shape the HPG axis prenatally and throughout childhood.

In conclusion for Study 2, the common genetic variance for autism did not correlate with the variance associated with steroid hormone levels in childhood. This may be because of low power in capturing the common genetic variance for autism, as well as the relatively low SNP-based heritability for postnatal hormone levels. However, the genetics of autism were found to correlate negatively with two complex, steroid-related traits, namely age of menarche and androgenic alopecia. The reason for this is unknown but likely involves a complex interaction between steroid hormones, the HPG axis and processes involved in cellular maturation. Additional research, both clinical and molecular, is needed to understand these novel correlations.

Finally, both the studies presented here indicate that genetic factors associated with autism, interact with the prenatal environment and particularly with the regulation of steroidogenesis via the placenta and the HPG axis. Further study is needed to understand if these interactions contribute substantially to the skewed sex ratios in neurodevelopment, particularly in comparison to social variables, such as gender norms and gender-based diagnostic biases for conditions like autism.

## Data availability statement

The original contributions presented in the study are included in the article/supplementary material. Further inquiries can be directed to the corresponding author.

## Author contributions

AT conducted the analysis, interpreted the data and drafted the manuscript. SB-C and VW contributed to study design, study supervision, data interpretation and to the revisions of the manuscript. All authors contributed to the article and approved the submitted version.
